# Defining the limits and indications of the Draf III endoscopic approach to the lateral frontal sinus and maximizing visualization and maneuverability: a cadaveric and radiological study

**DOI:** 10.1007/s00405-022-07323-9

**Published:** 2022-03-09

**Authors:** Efstathios Papatsoutsos, Aristotelis Kalyvas, Evangelos Drosos, Eleftherios Neromyliotis, Christos Koutsarnakis, Spyridon Komaitis, Vasileios Chatzinakis, George Stranjalis, Christos Georgalas

**Affiliations:** 1 Department of Otolaryngology, Head and Neck Surgery, University Clinic of Magdeburg, Otto von Guericke University of Magdeburg, Weber Str. 7, 39112 Magdeburg, Germany; 2 Division of Neurosurgery, Toronto Western Hospital/University Health Network, University of Toronto, Toronto, ON Canada; 3 Department of Neurosurgery, Evangelismos Hospital, National and Kapodistrian University of Athens, Athens, Greece; 4 Endoscopic Skull Base Centre Athens, Hygeia Hospital, Athens, Greece; 5 Medical School, University of Nicosia, Nicosia, Cyprus

**Keywords:** Lateral frontal sinus, Draf III, Cadaver, Piriform aperture, Orbital transposition

## Abstract

**Purpose:**

The DRAF III procedure has been used for access to the lateralmost part of the frontal sinus. We sought to identify anatomical and radiological measurements as well as modifications that predict the lateral limits of visualization and surgical access after this procedure.

**Methods:**

Seven cadaver heads were imaged with computed tomography scan. The distance from midline to the medial orbital wall (MOWD), midline to the lateral end of the frontal sinus (MLD), the sum of MLDs (SMLD), interorbital distance (IOD) and the shortest anteroposterior distance of the frontal recess (APD) were utilized. The ratios MLD/MOWD, and SMLD/IOD were calculated. The same distances were measured on 41 CT scans. Orbital transposition (OT) and partial resection of the piriform aperture (PAR) were performed; the visualization and reach were assessed. The angle of insertion was measured before and after the modifications.

**Results:**

Only the ratio MLD/MOWD was consistently predictive of access to the lateral, superior and posterior wall of the frontal sinus. Following the modifications, a visualization of 100% laterally was achieved with the 30- and 45 degree endoscopes and every lateral recess could be reached with the 70 degree suction. A mean increase of the angle of insertion of 25.3 and 59.6% was recorded after OT and PAR, respectively.

**Conclusions:**

IOD rather than APD defines the limits of the Draf III approach to the lateral frontal sinus and MLD/MOWD ratio can serve as a useful preoperative tool. Along to the already described OT, PAR increases visualization and reach of the lateral frontal sinus.

## Introduction

The endoscopic surgical approaches to the paranasal sinuses are rapidly evolving, leading to a continuous reconsideration of their limits, and broadening the spectrum of pathologies that can be treated endoscopically.

The management of lesions of the lateral frontal sinus constitutes a surgical challenge. The cornerstone of the endoscopic approaches to the lateral recess of the frontal sinus is the Draf III procedure. The Draf III procedure, also referred to as median drainage, was described by Draf et al. in 1991 [1]. This approach offers a wide surgical corridor to the frontal sinus. Nevertheless, the narrow frontal infundibulum, the acute angle from the nostrils to the sinus, the convex-shaped posterior and concave-shaped anterior sinus wall, the nasal beak as well as the medial and superior orbital wall pose substantial limitations to the endoscopic access to lateral frontal sinus.

Indications for an open or endoscopic procedure to the frontal sinus are not always clearly defined and depend on individual surgical expertise as well as localization of the pathology. The lack of robust data regarding the limits of the endoscopic approach obscures the decision-making process further. During the last few years, various authors assessed the limits of endoscopic frontal sinus surgery by operating lesions of the lateral frontal sinus beyond the virtual sagittal plane passing through the lamina papyracea. Endoscopic approaches to inverted papillomas [2–4], osteomas [5–7], mucoceles [8], Schwannomas [9] as well as CSF leaks [10–12], including lesions of the lateral frontal sinus [2, 6], have been described. Modifications of the Draf III procedure with partial removal of the lateral and superior orbital wall aim to increase lateral reach and instrument manoeuvrability [2, 13].

Becker et al. [14] assessed the level of visualization and reach to the frontal sinus after Draf IIA, IIB and III procedures and found that a Draf IIB and III procedure can extend the lateral limit of the endoscopic approach. Timperley et al. [15] estimated the lateral limit of the endoscopic access to the frontal sinus and the predictive value of variable CT scan measurements (shortest distance between the olfactory fossa and the inner and outer periosteum, the thickness of the nasofrontal beak, distance between the frontoethmoidal sutures and from midline to midorbital point). In an earlier study in osteomas [7], we suggested that using the wide access provided by a Draf III procedure and curved drills, it is possible to access the lateral supraorbital ridge well beyond the medial orbit. We proposed that it is neither the plane of lamina papyracea nor the 2 cm lateral to it that defines the lateral limits of resectability, but rather the ratio of lateral tumour extension to interorbital distance. Following removal of superior septum and drilling of the nasal beak, lateral access to the frontal sinus is restricted primarily by the orbital walls. In patients with relatively large interorbital distance, the potential lateral access is increased, whereas the opposite is true for narrow nasal inlet in the coronal plane. Lateral access to the floor of the frontal sinus (orbital roof) may, however, be limited [7].

The goal of this study is to evaluate the usefulness of different preoperative measurements and correlate them with the lateral limits of visualization and reach. Furthermore, we sought to assess the advantage offered by a partial resection of the medial and superior orbital wall as well as a partial transnasal resection of the piriform aperture in terms of access to the lateralmost part of the frontal sinus.

## Materials and methods

**Fig. 1:**
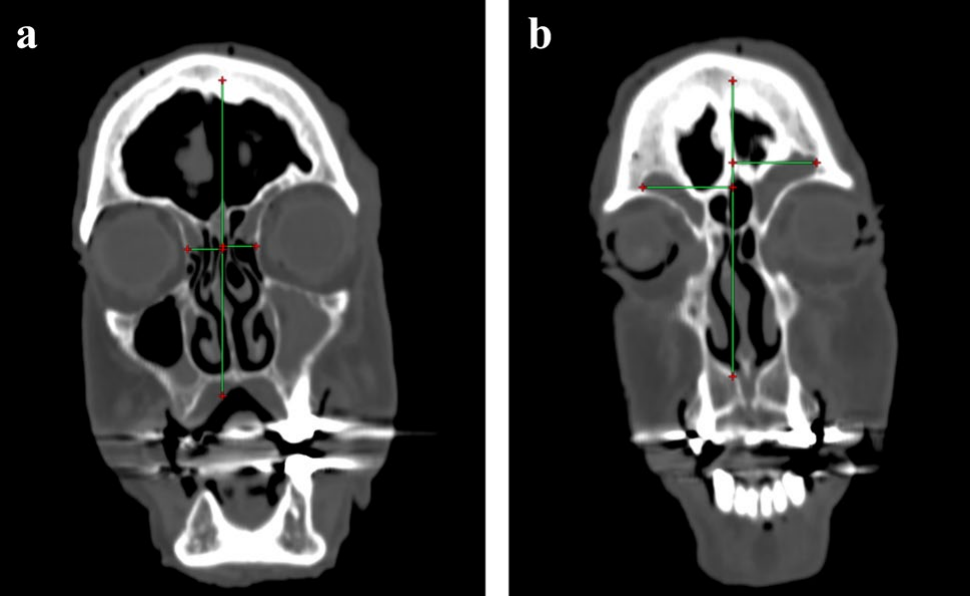
**a** Midline to medial orbital wall distance (MOWD), **b** Midline to lateral end of the frontal sinus distance (MLD)

A total of seven embalmed, fixed and injected cadaver heads (14 frontal sinuses) with no evidence of prior frontal sinus lesions, surgery or trauma were used in this study. They were imaged with thin cut, 1 mm computed tomography (CT scan). In order to produce comparable results, measurements of midline to the medial orbital wall (MOWD) for each side (Fig. 1a), interorbital distance (IOD = MOWD right + MOWD left), midline to the lateral end of the frontal sinus for each side (MLD) (Fig. 1b) and the sum of MLDs (SMLD) were performed on the coronal plane at the first slice posterior to the nasofrontal beak continuity, while for measurements of the shortest anteroposterior distance of the frontal recess (APD) sagittal images on each side on a plane centred on the ipsilateral olfactory fossa were utilized. Moreover, the pneumatization of the imaged frontal sinuses was classified into four zones (medial orbital wall, midway between medial orbital wall and midorbital point, midway between lateral orbital wall and midorbital point and lateral orbital wall) were measured. The ratios MLD/MOWD and SMLD/IOD were calculated. The same measurements (MOWD, IOD, MLD, APD) were performed on 41 CT scan of randomly selected patients with healthy paranasal sinuses. All measurements were carried out with the RadiAnt DICOM Viewer (Medixant, Poznan, Poland).

A Draf III procedure was performed on each head. A septal window was created, and the floor of the frontal sinus removed. The opening to the frontal sinus was maximized anteriorly by removing the nasofrontal beak until the anterior periosteum was reached and posteriorly to the first olfactory neuron. The heads were registered with a magnetic image guidance system (IGS) (FUSION Compact ENT Navigation System, Medtronic, Dublin, Ireland). On the next step the visualization of the lateral recess with a 0-, 30- and 45 degree endoscope and the reach of the lateral recess using 0- and 70 degree instruments was assessed. Additionally, the angle of insertion of a straight instrument to the sagittal plane was measured using the IGS and the GNU Image Manipulation Program (GIMP, The GIMP development team).

Subsequently, we performed two different modifications of the procedure to increase the lateral reach in the frontal sinus and to achieve greater freedom of movement. The first modification included the partial removal of the lateral and superior orbital wall (orbital transposition: OT). Firstly, the anterior ethmoid artery was identified. In a real surgical setting the coagulation and dissection of the artery would be necessary before proceeding with the orbital transposition. The lamina papyracea was fully exposed and thinned anteriorly and superiorly to the anterior ethmoid artery using a large diamond ball burr until the periorbita was reached. The thin bone layer was then carefully removed using a freer periosteal nasal elevator. Care was given not to detach the trochlea of the superior oblique muscle. The superior orbital wall was carefully removed using the 40 degree angled diamond drill as far and safe as possible, thus imitating a realistic operative attitude. During measurements the orbital contents were laterally transposed using a flexible retractor.

The second modification involves the transnasal partial resection of the piriform aperture (PAR). Again, the goal of this intervention is to increase the angle of instrument insertion into the frontal sinus. An incision was made in the transitional zone between skin and mucosa in the nasal valve area anterior and above the attachment of the inferior turbinate. The incision was carried down onto the edge of the piriform aperture. The periosteum was then elevated and the medial part of the anterior wall of the maxilla was resected. At this point, attention is needed to avoid an injury of the infraorbital nerve. Both interventions were performed in two heads each followed by a repetition of the measurements.

For the evaluation of the visualization and reach, the endoscopes and instruments were inserted via the contralateral nasal fossa. More specifically, endoscopes with three different angles (0, 30 and 45 degree) were used for the visualization of the lateral recess. The use of a 70 degree endoscope was deemed inappropriate for the detection of small differences in visualization as it provides an adequate view of the lateral recess in most of the cases, due to its wide angle. Reach of the lateral recess was defined as the contact of the instrument with the lateralmost wall of the frontal sinus while maintaining a certain degree of manoeuvrability and was evaluated using a straight and 70 degree suction/drill.

Statistical analysis was performed with SPSS statistics, version 26 (IBM, Armonk, NY). Correlations between CT measurements, such as the degree of pneumatization, APD, IOD, MLD and MOWD, were assessed with Spearman’s Rho correlation coefficient. *T* test was used to assess the factors impacting the reach and visualization of the lateral recess of frontal sinus, such as the ratios SMLD/IOD and MLD/MOWD.

## Results

A total of 14 frontal sinuses from seven heads were examined in this study. In 50% of the sinuses, the lateral recess extended beyond the midorbital point (zone 3), while the remaining half did not extend beyond the midorbital point (zone 2). No sinuses limited in zone 1 or extending to zone 4 were identified. In six out of seven heads, both frontal sinuses reached the same orbital zones. The initial measurements on the heads are summarized in Table 1. Table 2 demonstrates data regarding the visualization of the lateral frontal sinus after the Draf III procedure using 0-, 30- and 45 degree endoscopes.

**Table 1:** Baseline measurements on cadaver heads

CT scan orientation	Measurement	Left sinus (mean)	Right sinus (mean)
Sagittal	APD (anterior periosteum)APD (mm)	15	12.9
	Nasal beak (mm)	7.4	6.1
Coronal	MLD (mm)	29.6	27.5
	MOWD (mm)	13.1	12.7
	IOD (mm)	22.1	
	Ratio (MLD/MOWD)	2.27	2.17
	Ratio (SMLD/IOD)	2.62	
	Zones	2 (*n* = 3), 3 (*n* = 4)	2 (*n* = 5), 3 (*n* = 2)

**Table 2:** Visualization with 0-, 30- and 45 degree endoscopes

Sinus border	Left sinus			Right sinus		
	0° (%)	30° (%)	45° (%)	0° (%)	30° (%)	45° (%)
Anterior	42.9	85.7	100	42.9	85.7	100
Posterior	100	100	100	100	100	100
Superior	100	100	100	100	100	100
Lateral	0	57.1	85.7	0	85.7	100

The specimens where the anterior wall of the frontal sinus could be visualized with a 0 degree endoscope had longer APD (mean 9 mm vs 5.9 mm, *t*(12) = − 3.4, *p* = 0.005), while if that could be achieved with a 30 degree endoscope had lower SMLD lengths (mean 5.5 vs 7.2 mm *t*(5) = 1.8,*p* = 0.13 for either right or left sinus visualization), lower MLD lengths (mean 2.7 vs 3.6 mm, *t*(12) = − 2.5, *p* = 0.03) and lower MLD/MOWD ratios (mean 2.1 vs 2.8, *t*(12) = − 2.8, *p* = 0.017) compared to specimens with no obtainable visualization.

The percentages of sinuses reached with the straight and 70 degree angled instruments are summarized in Table 3. Specimens where a 70 degree instrument could reach the superior, posterior and lateral wall of the frontal sinus featured lower MLD/MOWD ratios (mean 2.1 vs 2.6, *t*(12) = 2.7, *p* = 0.019 for superior wall access; mean 2.1 vs 2.8, *t*(12) = 2.8, *p* = 0.017) for posterior wall access; mean 2.1 vs 2.8, *t*(12) = 2.8, *p* = 0.017) for lateral wall access), lower MLD lengths (mean 2.6 vs 3.5 mm, *t*(12) = 4, *p* = 0.002 for superior wall access; mean 2.7 vs 3.6 mm *t*(12) = 2.5, *p* = 0.03 for posterior wall access; mean 2.7 vs 3.6 mm, *t*(12) = 2.5, *p* = 0.03) for lateral wall access) and lower SMLD (mean 5.2 vs 6.9 mm, *t*(5) = 3.1, *p* = 0.03 for superior wall access; mean 5.5 vs 7.1 mm, *t*(5) = 1.8, *p* = 0.13 for posterior wall access; mean 5.5 vs 7.1 mm, *t*(5) = 1.8, *p* = 0.13, for either right or left sinus reach) compared to samples with non-obtainable reach.

**Table 3:** Reach with straight and 70 degree instrument

Sinus Border	Left sinus		Right sinus	
	0° (%)	70° (%)	0° (%)	70° (%)
Anterior	0	100	0	100
Posterior	100	85.7	100	85.7
Superior	57.1	71.4	57.1	71.4
Lateral	0	85.7	0	85.7

**Fig. 2:**
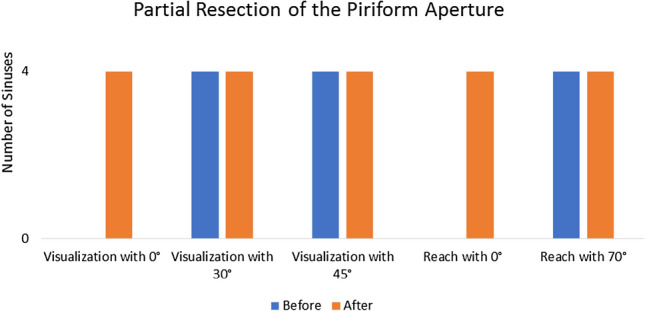
Changes in visualization and reach after partial resection of the piriform aperture

Following PAR on two heads (four sides), the visualization of the lateral recess with the 0 degree endoscope became possible in all four sinuses. Visualization with the 30- and 45 degree endoscope was achieved with more ease. The access to the lateral end of the frontal sinus with the straight suction also became possible in every sinus (4/4). The insertion of the 70 degree instrument was possible with less resistance and the freedom of movement increased (Fig. 1). An increase of the angle of insertion, measured with the IGS using the straight instrument, was revealed in every case (Table 2). More specifically, a mean increase of 59.6% was recorded. In a different group of two heads the Draf III procedure was modified through a bilateral OT. This led to the visualization of the sinus lateral wall in one right sinus, which in three cases remained invisible to the 0 degree endoscope despite the improvement of the angle. When the 30- and 45 degree endoscopes were used, all four sinuses could be visualized until their lateral end. OT made the access to the lateral recess with the 0 degree suction possible in one sinus and improved it in the rest of the sinuses. The reach with the 70 degree instrument was improved from 50% (2/4 sinuses) before to 100% (4/4 sinuses) after the adjunct surgical step (Fig. 2). Moreover, using the straight instrument an increase of the angle of insertion (mean increase of 25.3%) was revealed in every case (Table 4). Overall, after the above aforementioned modifications to the Draf III procedure a visualization of 100% laterally was achieved with the 30- and 45 degree endoscopes and every lateral recess could be reached with the 70 degree suction. Furthermore, the PAR allowed a lateral reach in 100% of the examined sinuses with the straight suction. The baseline results of the measurements on the 41 CT scans are summarized in (Table 5).

**Fig. 3:**
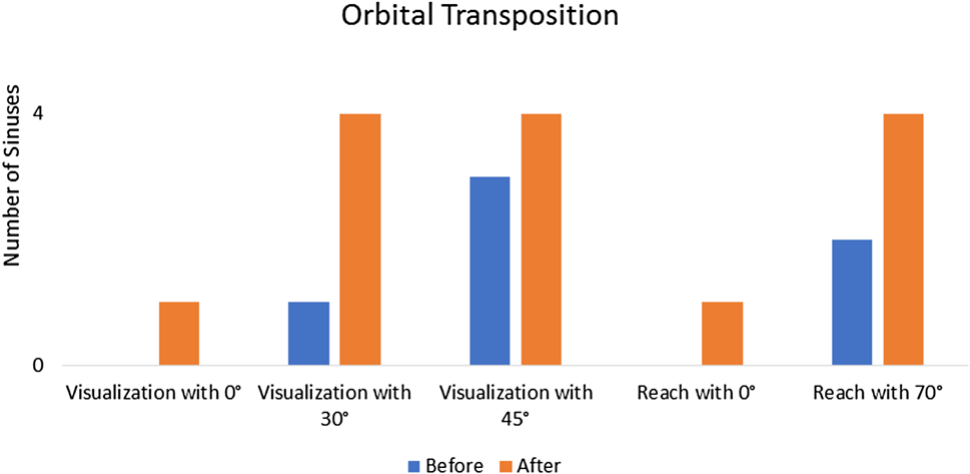
Changes in visualization and reach after orbital transposition

**Table 4:** Change of the angle of insertion after the modifications of Draf III procedure

Partial resection of the pyriform aperture	Increase of angle of insertion (%)
Head 1-left frontal sinus	10.12 (40.5)
Head 1-right frontal sinus	14.87 (140.5)
Head 2-left frontal sinus	11.93 (36.5)
Head 2-right frontal sinus	10.10 (81.2)
Orbital transposition	
Head 3-left frontal sinus	10.75 (51.7)
Head 3-right frontal sinus	1.93 (10.2)
Head 4-left frontal sinus	13.01 (35.4)
Head 4-right frontal sinus	6.50 (36.4)

**Table 5:** Baseline measurements on random head CT scans

CT scan orientation	Measurement	Left sinus (mean)	Right sinus (mean)	
Sagittal	APD (mm)	15.5	15.5	
	Nasal beak (mm)	9.9	9.6	
Coronal	MLD (mm)	30.4	30.5	
	MOWD (mm)	10.9	11.2	
	IOD (mm)	23.4		
	Ratio (MLD/MOWD)	2.84	2.76	
	Ratio (SMLD/IOD)	2.61		
	Zones	1(2), 2(16), 3(20), 4(3)	1(2), 2(22), 3(16), 4(1)	

The statistical analysis of the measurements on the 41 CT scans revealed a significant correlation of MOWD with MLD (*R* = 0.34, *P *= 0.002), nasofrontal beak thickness (*R* = 0.366, *p* = 0.001) and APD of frontal ostium (*R* = 0.29, *p* = 0.008). Not unexpectedly, the degree of pneumatization of the frontal sinus was very strongly correlated with the MLD (*R* = 0.78, *p* < 0.0001). The significant correlations of the anatomical measurements are shown in (Fig. 3).

**Fig. 4:**
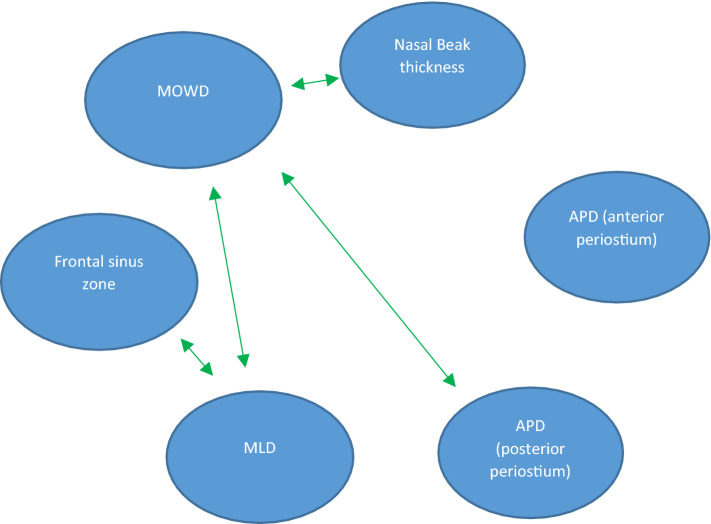
Significant correlations between the anatomical CT-measurements

## Discussion

Typically, the discussion on the indication and limitation of endoscopic frontal sinus surgery has focused on the thickness of nasofrontal beak and the anteroposterior distance of frontal recess [16–21]. It has been widely accepted that Draf III was either unlikely to be successful or was contraindicated in patients with short anteroposterior distance of the frontal recess–which most of the times was associated with a thick nasofrontal beak. However, the modifications of Draf III in the recent years have aimed to improve the reach and effectiveness of the procedure by focusing on the limitations posed by the orbits on the coronal plane rather than on the anteroposterior (sagittal) axis. Our longstanding surgical observation that the limited space on the coronal level (between the orbits) rather than sagittal level poses a greater limitation to the lateral reach was the hypothesis behind this anatomical and cadaveric study.

The present anatomical study ought to evaluate some of the commonly used preoperative CT measurements (APD, nasofrontal beak thickness) and clarify the value of others (MOWD, IOD, MLD, SMLD, MLD/MOWD, SMLD/IOD) in predicting the endoscopic accessibility of the frontal sinus following Draf III. The radiological part was important to assess the correlations between the various measurements and their relative value as independent predictors, rather than as secondary associations.

The most crucial part of operating in the frontal sinus (for example, for drilling the attachment of an inverted papilloma or an osteoma) is the reach of a curved (usually 70 degree) instrument or drill. Only the ratio MLD/MOWD was consistently strongly predictive of access to the lateral, superior and posterior wall of the frontal sinus with the 70 degree instrument. This observation underlines the potential usefulness of these measurements to predict the endoscopic accessibility of lesions located in the lateral portion of the frontal sinus. Unfortunately, a clear cutoff point could not be identified. Interestingly, the correlation of the endoscopic reach with the APD (anterior or posterior periosteum or the nasofrontal beak) did not reach significance.

To summarize, there are two crucial factors related to the anatomy of the sinus system that can influence endoscopic operability. The first is the angle of insertion on the coronal plane. The two anatomical points that mainly confine an inserted instrument are the pyriform aperture and the orbital wall. More specifically, the reach of the far lateral frontal sinus and the freedom of motion in the lateral position is limited by the contralateral lateral border of the pyriform aperture and the unilateral superomedial orbital wall. The second factor includes the manoeuvrability in the sagittal plane, which is mainly depending on the APD. Particularly for the anterior and posterior sinus wall, APD constitutes an important preoperative measurement. In this context, a prerequisite to maximize freedom of instrument movement and hence the overall accessibility of the sinus is the complete drilling of the nasofrontal beak. Consequently, the MLD and MOWD as well as the MLD/MOWD ratio, when combined with the APD, offer substantial information to support the decision for an endoscopic approach. Anatomical variants with a prominent posterior sinus wall and a very concave orbital roof, that obscures the view of the caudal portion of the lateral recess, constitute additional factors with great influence on the accessibility of the frontal sinus and should not be ignored.

During the second part of this anatomical study, we evaluated the effect of two different modifications to the Draf III on the endoscopic accessibility of the lateral part of the frontal sinus. A statistical analysis of the results was not performed, because of the small number of sinuses studied. The results regarding both surgical interventions identified a substantial improvement of visualization and reach of the lateral part of the frontal sinus, which in case of the OT are in concordance with the existing literature [2, 13]. The endoscopic PAR has, in our knowledge, never been described as an adjunct surgical step to improve the access to the frontal sinus. These kind of procedures are described by Woodhead and Smith et al. [22, 23] in the context of the surgical treatment of alar collapse and the improvement of nasal airway. Additionally, this modification is part of the technique described by Alfred Denker [24] (who used a sublabial corridor) and by Sturmann and Canfield [25, 26] (who described an endonasal approach) with the goal of increasing the exposure to the maxillary sinus. Battaglia et al. [27] and Upadhyay et al. [28] used this surgical step to increase visualization of and instrument manoeuvrability in the infratemporal fossa, the parapharyngeal space and the pterygopalatine fossa.

The effect was stronger after the endoscopic PAR. However, a visualization of the lateral recess with the 30 degree endoscope was possible in all four sinuses before the PAR, while only one lateral recess was visible before the OT. This difference may have contributed to the greater angle change after the partial resection of the piriform aperture. In both interventions, a reduction of the angle between the horizontal axis of the frontal sinus and that of instrument insertion leads to a better reach and an improved freedom of motion in the sinus and especially its far lateral portion. Exposure of lateral lesions obscured by a prominent posterior sinus wall is not expected to improve significantly with these adjuncts. Moreover, the reach of the far lateral frontal sinus in cases with an extremely convex orbital roof will improve after OT. In those patients, no substantial benefit is expected from a PAR.

Both interventions were studied only after the performance of a Draf III procedure. Although they comprise simple surgical steps, they need to be performed with caution to avoid damage of the neighbouring structures. During the partial endoscopic PAR care should be taken not to damage the infraorbital nerve. Additionally, a significant transposition of the nose to the side of the resection is theoretically possible through the inserted instruments after expansion of the piriform aperture, as the diameter of the nasal nostrils remained unchanged. This could lead to soft tissue injuries or haematomas in a real surgical setting. On the whole, resecting parts of the pyriform aperture to improve endoscopic access may “defeat the purpose” by transforming an endoscopic procedure to a more invasive, complicated and more time consuming one. Alternatively, a transfacial procedure can be preferred in cases with limited endoscopic visualization and reach of the lateral frontal sinus. In this study the theoretical feasibility of the PAR was explored, although it cannot be proposed as a standard appendix to a Draf III procedure. In any case, the surgeon must always balance the pros and cons of endoscopic and open procedures to offer the best possible treatment depending on the individual anatomy and the type of the disease treated, regardless of their preferences.

In the case of OT, following removal of orbital bony wall, there is a theoretical risk of orbit expansion. However, orbital periosteum forms a layer strong enough to prevent this, and indeed, in orbital decompression, it is the orbital periosteal incision that signals the initiation of decompression. In the studies performed and referenced, a minor expansion of the orbit was noted, but it was not significant enough to narrow the ostium [2, 13].

## Conclusion

The endoscopic approach for lesions of the far lateral frontal sinus is a viable surgical option. This study identified the MLD/MOWD ratio as a useful preoperative tool to facilitate decision making when an endoscopic approach is considered for a lesion in the lateral frontal sinus. Our measurements reveal a constant correlation of this ratio with the instrument reach in the frontal sinus. A similar correlation could not be identified for the traditional measurements on the sagittal plane. This fact underlines the value of this measurement and suggests that its routine use in the preoperative setting may substantially facilitate decision making. Furthermore, we propose, along to the already described OT, PAR as an adjunct surgical step to increase visualization and reach of the far lateral frontal sinus. The main limitation of the present study is its small group of sinuses, which in combination with the significant variability in the degree of pneumatization and size of the frontal sinus underlines the need for additional studies with a greater number of sinuses to confirm or reject our findings and to assess the effect of anteroposterior supraorbital pneumatization on the degree of access. However, the fact that there was a consistent statistical association between the measured ratio and surgical access indicates that it is a potentially important measurement. Setting a cutoff value for the visualization and reach of the lateral part of the frontal sinus as well as the utilization of the adjunct surgical steps may also be a field of future study.
